# (+)-Discodermolide: A Marine Natural Product Against Cancer

**DOI:** 10.1100/tsw.2004.96

**Published:** 2004-06-11

**Authors:** Marcus Viníus Nora De Souza

**Affiliations:** FioCruz-FundaÇão Oswaldo Cruz, Instituto de Tecnologia em Fármacos-Far Manguinhos, Rua Sizenando Nabuco, 100, Manguinhos, 21041-250 Rio de Janeiro-RJ, Brazil

**Keywords:** (+)-discodermolide, natural products, cancer, tubuline

## Abstract

(+)-Discodermolide was isolated in 1990 by Gunasekera et al. from the deep-water Caribbean sponge Discodermia dissoluta. It attacks cancer cells in a similar way to the successful cancer drug Taxol® that has become the best-selling anticancer drug in history. Taxol is also the first natural product described that stabilizes the microtubules involved in many aspects of cellular biology and that represent an important target of anticancer chemotherapeutics. However, (+)-discodermolide appears to be far more potent than Taxol® against tumors that have developed multiple-drug resistance, with an IC_50_ in the low nanomolar range. Due to these excellent results, this natural product was licensed to Novartis Pharmaceutical Corporation in early 1998. The present review covers the history, biological activity, total synthesis, and synthetic analogs of (+)-discodermolide.

## INTRODUCTION

Cancer is a growing public health problem with estimated worldwide new cases at over a million per year[1]. In the U.S., cancer is the second leading cause of death after heart disease, but it is the major cause of death in women between the ages of 35 and 74[2]. If the current trend continues, cancer is expected to be the primary cause of death in the U.S. by the year 2010[2]. Although cancer is often referred to as a single condition, it actually consists of more than 100 different diseases. These diseases are characterized by uncontrolled growth and spread of abnormal cells. All the organs in the body (such as the lung, breast, colon, and brain) consist of specialized cells that carry out the organ's functions, such as transport of oxygen, digestion of nutrients, excretion of waste materials, locomotion, reproduction, and thinking. Cancer can arise in many sites and behave differently depending on its organ of origin[3]. In general, cancer falls into four major groups, classified according to the body tissues in which they arise. All types can spread to other kinds of body tissues, while retaining their original cellular characteristics.

- *Carcinomas:* tumors that begin in epithelial tissue, the cells that form the outside surface of the body and line the inner passages
- *Sarcomas*: tumors originating in the connective tissues, principally the muscles, bones, cartilage, and connective tissues
- *Lymphomas:* tumors in the lymphatic system
- *Leukemias:* cancers of the blood, forming tissue

Considerable progress has been made towards controlling several forms of cancer through a combination of prevention and therapy, but some types of solid tumors such as melanoma, ovarian, breast, and lung, in particular, remain a major issue of both scientific and public concern. One of the most promising antitumor agents developed over the past 3 decades is paclitaxel (Taxol®) (Fig. 1), a natural product extracted from the bark of the Pacific Yew, *Taxus brevifolia Nutt*[4]. Taxol® is a member of the taxane family of diterpenes, which currently has over 300 components isolated from natural sources[5]. Taxol® was approved by the FDA for the treatment of refractory ovarian cancer in December 1992 and was introduced into the marketplace in January 1993 by Bristol-Myers Squibb Company (BMS) operating under a Cooperative Research and Development Agreement (CRADA) with the National Cancer Institute (NCI)[6]. Approval for treatment of breast cancer followed in 1994[7]. Since then, the clinical use of Taxol® has increased steadily and at present it is used not only for treatment of ovarian and breast cancers, but also for treatment of both small-cell and nonsmall-cell lung cancer, squamous cancer of the head and neck, as well as various others kinds of cancer[5]. It is currently available in more than 60 countries. Worldwide sales of Taxol® (Fig. 1) reached $1.6 billion in 2000[8] and it has become the best-selling anticancer drug in history.

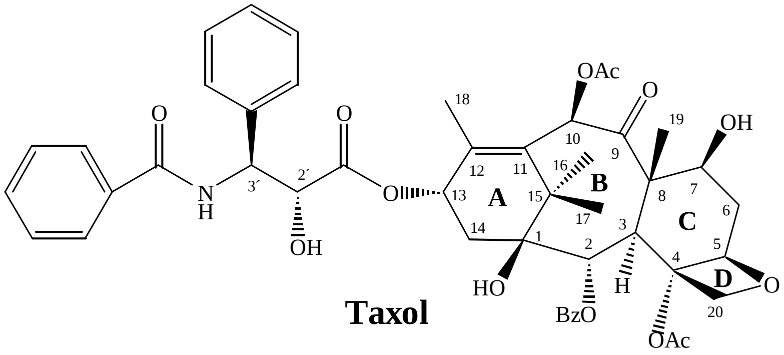

**FIGURE 1**

The success story of Taxol® has demonstrated once again the power of Mother Nature in terms of biologically active molecules as a cure for disease. The search for new compounds with a mechanism of action similar to that of Taxol® (Fig. 1), but with greater aqueous solubility and efficacy, particularly in Taxol-resistant cells, has led to the isolation of new natural products[9]. After Taxol®, other natural products have been found to bind and stabilize cellular microtubules[9,10,11], such as, epothilone A and B[12], eleutherobin and sarcodictyin A[13], (–)-laulimalide[14], and FR182877[15] (Fig. 2).

Another promising natural product microtubule-stabilizing anticancer agent is (+)-discodermolide (Fig. 3). This compound, derived from a deep-sea sponge, shows potent antitumor activity and holds great promise in the development of a drug for the treatment of cancer. The history, biological activity, synthesis, and synthetic analogs of (+)-discodermolide will be covered in this review.

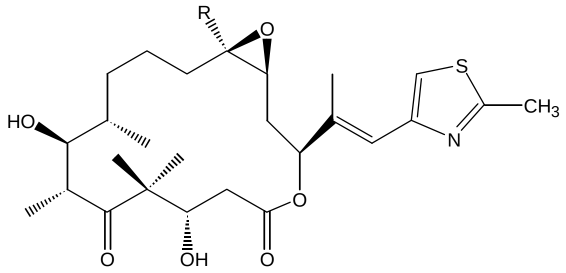

**R = H Epothilone A**
**R = Me Epothilone B**

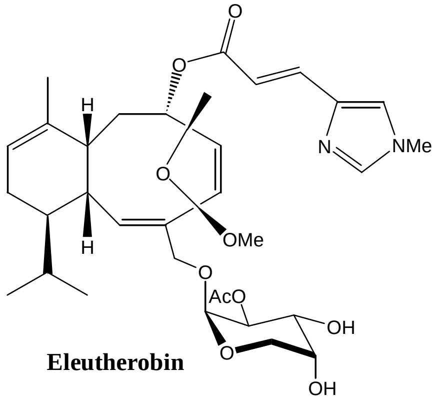

**Eleutherobin**

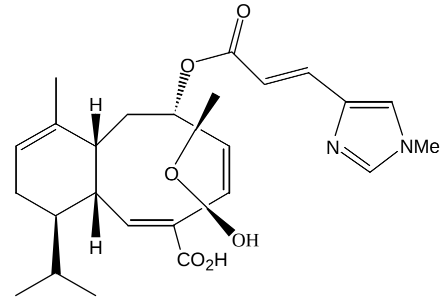

**Sarcodictyin A**

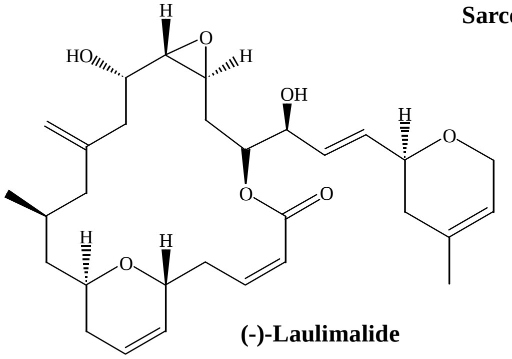

**(-)-Laulimalide**

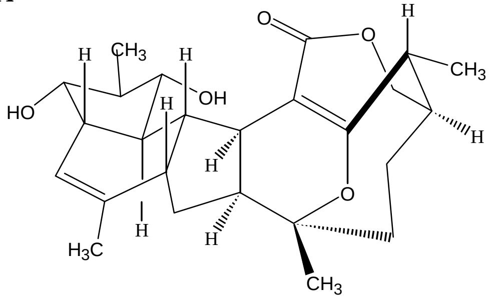

**WS9885B**

**FIGURE 2**

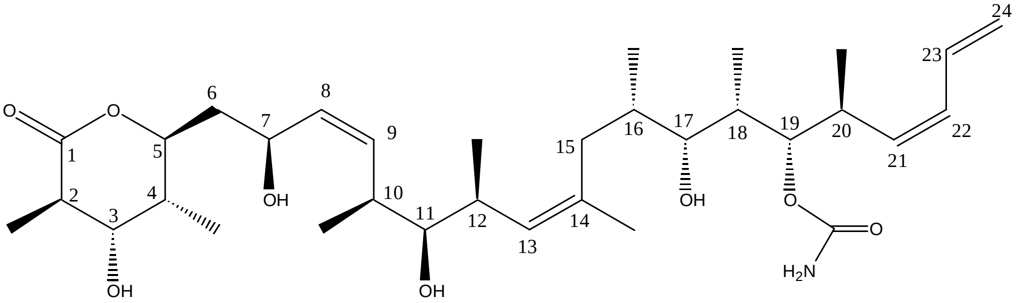

**(+)-Discodermolide**

**FIGURE 3**

## A BRIEF HISTORY OF THE (+)-DISCODERMOLIDE

In the 1990s, the discovery of a new cytotoxic polyketide natural marine product began to unfold. The source of (+)-discodermolide (Fig. 3), the deep-water Caribbean sponge *Discodermia dissolute*, was first collected by Pomponi et al.[16] of the Biomedical Marine Research division at the Harbor Branch Oceanographic Institute (HBOI) in 1987[17]. Subsequently, (+)-discodermolide was isolated and characterized by Gunasekera et al. at HBOI and its gross structure was determined by extensive spectroscopic studies and the relative stereochemistry was assigned by single-crystal X-ray crystallography[17]. The absolute configuration, however, remained undefined until the Schreiber group synthesized both antipodes[18]. Structurally, it bears 13 stereogenic centers with 15 rotatable σ bonds, a tetrasubstituted δ-lactone ($C_1$-$C_5$), one di ($C_8$-$C_9$) and one trisubstituted ($C_{13}$-$C_{14}$) (Z)-double bond, a pendant carbamate moiety ($C_{19}$), and a terminal (Z)-diene ($C_{21}$-$C_{24}$). The solution structure of (+)-discodermolide was determined by the Smith group[19] using 1 and 2-D NMR techniques in conjugation with Monte Carlo conformational analysis. This work demonstrated, as well as the crystal structure demonstrated, that this natural product exists in a helical conformation with the C1-C19 region in a U-shape conformation, bringing the δ-lactone and the C19 side chain in close proximity. The Synder group[20] also described the solution structures of (+)-discodermolide in a high-field 2-D NMR analysis followed by a NAMFIS analysis (NMR analysis of molecular flexibility in solution) in DMSO and showed similar results to those of Smith et al.

In subsequent studies with his collaborators, Longley[21] found that (+)-discodermolide (Fig. 3) was a potent immunosuppressive agent, both *in vivo* and *in vitro*. In 1994, the Schreiber group[22] showed distinct binding and cellular properties of synthetic (+) and (–)-discodermolide. However, in 1996, biological screening of this compound revealed that this substance kills tumor cells through induction of tubulin polymerization to microtubules and microtubule stabilization[23]. (+)-Discodermolide has been found to stabilize microtubules more potently than Taxol® and appears to be far more powerful than Taxol® against Pgp-expressing multiple drug resistant (MDR) cells (PgP = P-glycoprotein), with an $IC_{50}$ in the low nanomolar range[24]. (+)-Discodermolide has also been predicted to be 100-fold more soluble than Taxol®. (+)-Discodermolide (Fig. 3) represents a leading compound for a new class of microtubule-stabilizing agents with activities that may prove therapeutically useful for the treatment of Taxol-resistant breast, ovarian, and colon cancer and multidrug-resistant cancers[25]. These observations gave rise to a great deal of excitement, anticipating the possible development of this compound as a cancer agent, particularly in view of its effectiveness against a number of taxol-resistant tumor cell lines. Due to these excellent results, (+)-discodermolide (Fig. 3) was licensed to Novartis Pharmaceutical Corporation (Basel, Swizerland) in 1998 for development as an anticancer drug and is in phase-I clinical studies. The present review covers the biological activity of this fascinating natural product and its synthetic analogs and discusses their potential in cancer chemotherapy.

## BIOLOGICAL PROPERTIES OF (+)-DISCODERMOLIDE

Initial investigations of the biological properties of (+)-discodermolide (Fig. 2) focused on its action as an immunosuppressive agent[17,21]. As an immunosuppressive agent, it has an $IC_{50}$ of 9 n*M* for inhibition of purified murine T cell proliferation, inhibits the mixed leukocyte reaction, and suppresses graft-versus-host disease in transplanted mice. Further, this compound has also been shown to cause cell cycle arrest in the gap2 (G2) or mitosis (M) phase in a variety of human and murine cell lines, with $IC_{50}$ values ranging from 3 to 80 n*M*[25]. The growth of Taxol-resistant ovarian and colon cancer cells is inhibited by (+)-discodermolide with an $IC_{50}$ < 2.5 n*M*[24], while the timing and type of DNA fragmentation induced is consistent with the induction of apoptosis[26]. Investigation also revealed that the mode of action of (+)-discodermolide is similar to that of the anticancer agent Taxol® that stabilizes microtubules[23].

## (+)-Discodermolide and Microtubules

Tubulin plays an important role in the cell cycle, particularly during mitosis. Tubulin is a heterodimeric protein composed of globular $\alpha$ and $\beta$ tubulin subunits with approximately 440 amino acids each. Both forms have a molecular mass of approximately 50 kDa and a diameter of about 4 to 5 nm that polymerizes into long chains or filaments that form microtubules[27]. Microtubules are proteinaceous elements found in nearly all eukaryotic cells and can be characterized as long hollow cylinders with outer diameters of between 20 and 30 nm and an inner diameter of about 13 nm, which serve as a skeletal system for living cells[28]. In cooperation with other components of the cytoskeleton, specifically with actin microfilaments and intermediate filaments, microtubules are involved in several basic cellular processes such as segregation of genetic material, intracellular transport, maintenance of cell shape, positioning of cell organelles, extracellular transport by means of cilia, and movement of cells by means of flagella and cilia[12]. Microtubules have the ability to shift through various formations, which permits a cell to undergo mitosis or to regulate intracellular transport. The formation shifting of microtubules is made possible by the flexibility of tubulin, which is why scientists have been seeking to understand the protein's atomic structure since its discovery in the 1950s[29]. Because of their versatility, usage, and importance to growing cells, microtubules have been called “the most strategic subcellular targets of anticancer chemotherapeutics”[6].

(+)-Discodermolide (Fig. 3) is an antimitotic compound that causes an irreversible disorganization of microtubules, which results in the disruption of cellular division and induction of apoptosis[30]. Its mechanism of action showed potent induction of tubulin assembly and stabilization of microtubules[23]. When compared to Taxol® (Fig. 1), (+)-discodermolide was found to be more potent at nucleating tubulin assembly, at inducing microtubule bundles in MCF-7 cells, and to have a higher affinity for tubulin. For example, when the drug was added to a tubulin solution *in vitro,* assembly occurred instantaneously, indicating a major effect on the initiation and nucleation processes of microtubule assembly[23]. The Day group has shown that discodermolide-treated breast carcinoma cells display a spectacular rearrangement of the microtubule cytoskeleton, including extensive microtubule bundling. The same microtubule rearrangement that occurred with 10-n*M* (+)-discodermolide required 1 $\mu M$ Taxol®[23]. (+)-Discodermolide had equally impressive effects on tubulin assembly *in vitro*. Near-total polymerization occurred at 0ºC with tubulin plus microtubule associated proteins (MAPs) with or without GTP, and tubulin assembly was more vigorous with discodermolide than with Taxol® under every condition examined. (+)-Discodermolide-induced polymer differed from Taxol-induced polymer in that it was completely stable at $0^{o}$C in the presence of high concentrations of $Ca^{+2}$. In a quantitative assay designed to select for more effective agents than taxol in inducing assembly, discodermolide had an $EC_{50}$ value of 3.2 $\mu M$ vs. 23 $\mu M$ for Taxol®[23].

## Synergism between Taxol® and (+)-Discodermolide

Although Taxol® has had clinical success as a single agent and in combination with cisplatin[31], its use in combination with other antitumor agents is now under intense evaluation, particularly for the treatment of advanced or recurrent cancers that are refractory to standard chemotherapy[32]. Many combination therapies now being tested use drugs with similar mechanisms of action. In comparative studies of (+)-discodermolide (Fig. 3) with the epothilones and eleutherobin (Fig. 2) against a Taxol-dependent human lung carcinoma cell line (A549-T12)[49], it was found that (+)-discodermolide was unable to act as a substitute for Taxol® (Fig. 1), whereas the epothilones and eleutherobin (Fig. 2) were able to maintain the viability of the cell line. Significantly, the presence of low concentrations of Taxol® amplified the cytotoxicity of (+)-discodermolide 20-fold against this cell line[33] and suggests that Taxol® and discodermolide may constitute a promising chemotherapeutic combination[34]. However, this synergistic effect *in vitro* was not observed with combinations of the epothilones or eleutherobin (Fig. 2) with Taxol® (Fig. 1).

## THE ISSUE OF SUPPLY.

Discodermolide (Fig. 3) is one of several bioactive molecules that are naturally produced by marine organisms such as sponges, tunicates, soft coral, and microbes. However, the supplies of these compounds are only available in limited quantities from their natural sources. The natural sponge population would not be able to survive the massive collection needed to meet the testing and production demands of the pharmaceutical industry for discodermolide. For example, the reported isolation yield of (+)-discodermolide is only 0.002% (w/w from frozen sponge), the equivalent of only 7 mg of natural product from 434 g of sponge[17]. Due to this problem, researchers followed up on the discovery by focusing on creating alternative ways of producing this natural product. At present, total synthesis provides the only viable means of accessing useful quantities of (+)-discodermolide (Fig. 3) for clinical development[35]. A good example is the total synthesis realized by the Smith group[36], which has been utilized to produce (+)-discodermolide on a gram scale and the large-scale synthesis realized by Mickel et al. that has been used as a hybridized Novartis-Smith-Paterson synthetic route to produce this natural product on a 60-g scale[37].

## DEVELOPMENT OF TOTAL SYNTHESIS OF DISCODERMOLIDE

The Schreiber group was the first to synthesize (+)-discodermolide[18a] (Fig. 3) and its antipode, (–)-discodermolide[18b] (Fig. 4), establishing the absolute configuration and preparing a number of structural analogs[18]. After that, several other groups have synthesized (+)-discodermolide[35,36,37,38,39,40], the antipode (–)-discodermolide[36d,37], and various fragments of discodermolide, using different synthetic approaches[41]. The aforementioned groups focus on the preparation and evaluation of natural and synthetic analogs of discodermolide, which may lead to a better understanding of the structure-activity relationship (SAR) of (+)-discodermolide[18,42], as well as to an improvement in the efficiency of the synthesis of the compound. This could ultimately lead to a drug that would be more powerful and easier to synthesize than the parent compound, (+)-discodermolide.

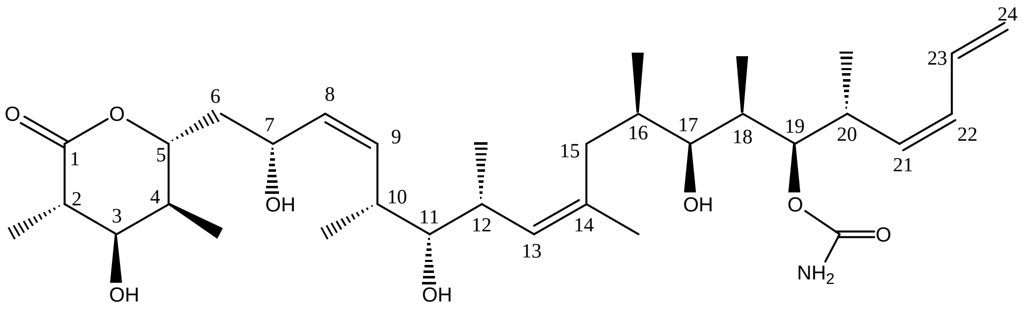

**(-)-Discodermolide**

**FIGURE 4**

## Schreiber Synthesis of Discodermolide

The design of the total synthesis of the Schreiber group was based on a convergent approach to discodermolide[18b] (Scheme 1). Retrosynthetically, this natural product was divided into three fragments — **1** (C1-C7), **2** (C8-C15), and **3** (C16-C24) — that were produced from the homoallylic alcohols **4** and **5** obtained from the chiral aldehyde **6**, derived from the (+)-3-hydroxy-methylpropionate methyl ester **7**. The Schereiber synthesis of (+)-discodermolide involved two key fragment couplings at (C7-C8) and (C15-C16), based on a Nozaki-Kishi addition reaction[43] and an enolate alkylation, respectively (Scheme 1). This synthesis provided the (+)-discodermolide in 36 steps with an overall yield of 4.3% over 24 steps (in the longest linear sequence). The important contributions of the Schreiber synthesis of (+)-discodermolide were the unambiguous assignment of the absolute stereochemistry of this natural product and the preparation of different analogs, which provided the first SAR study. Also, the synthesis and discovery that the unnatural antipode (–)-discodermolide (Fig. 4) is also cytotoxic and causes cell cycle arrest in the S-phase[18a].

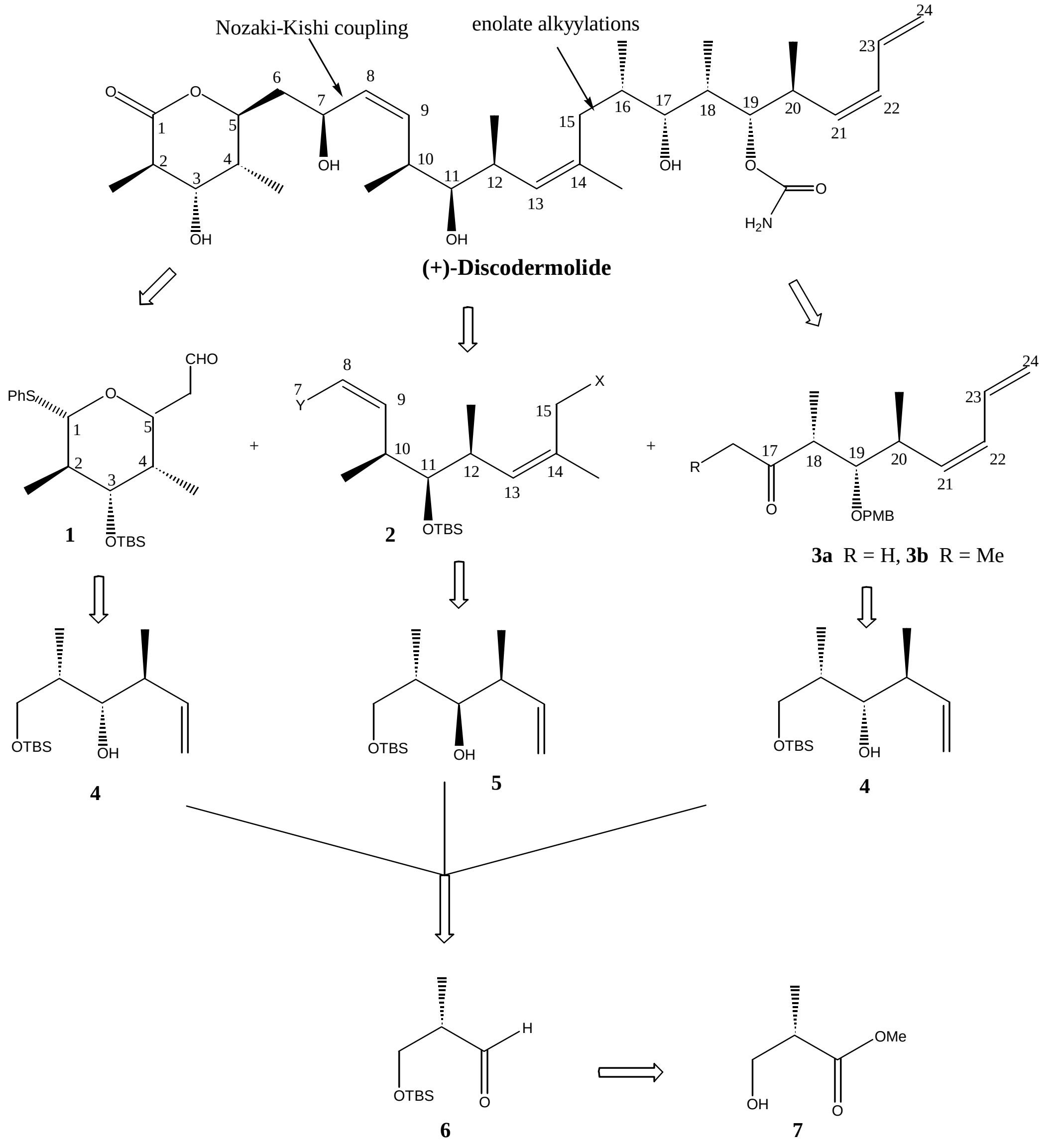

**SCHEME 1**

## Smith Synthesis of Discodermolide

Smith et al. first achieved the total synthesis of *ent*-discodermolide based on a triply convergent approach, which provided the *ent*-discodermolide with an overall yield of 2.2% over 28 steps (in the longest linear sequence)[36d]. Subsequently, Smith et al. (using the same triply convergent approach) developed a second-[36b,c] and third-generation[36a] synthesis both reducing the number of steps and yielding the discodermolide on a gram scale. The strategy of Smith's second-generation synthesis involved key fragment couplings at (C8-C9) and (C14-C15) **8-10** using a Wittig olefination and a Negishi cross-coupling reaction, respectively (Scheme 2). This total synthesis provided the (+)-discodermolide with an overall yield of 6.0% over 24 steps (in the longest linear sequence)[36b,c].

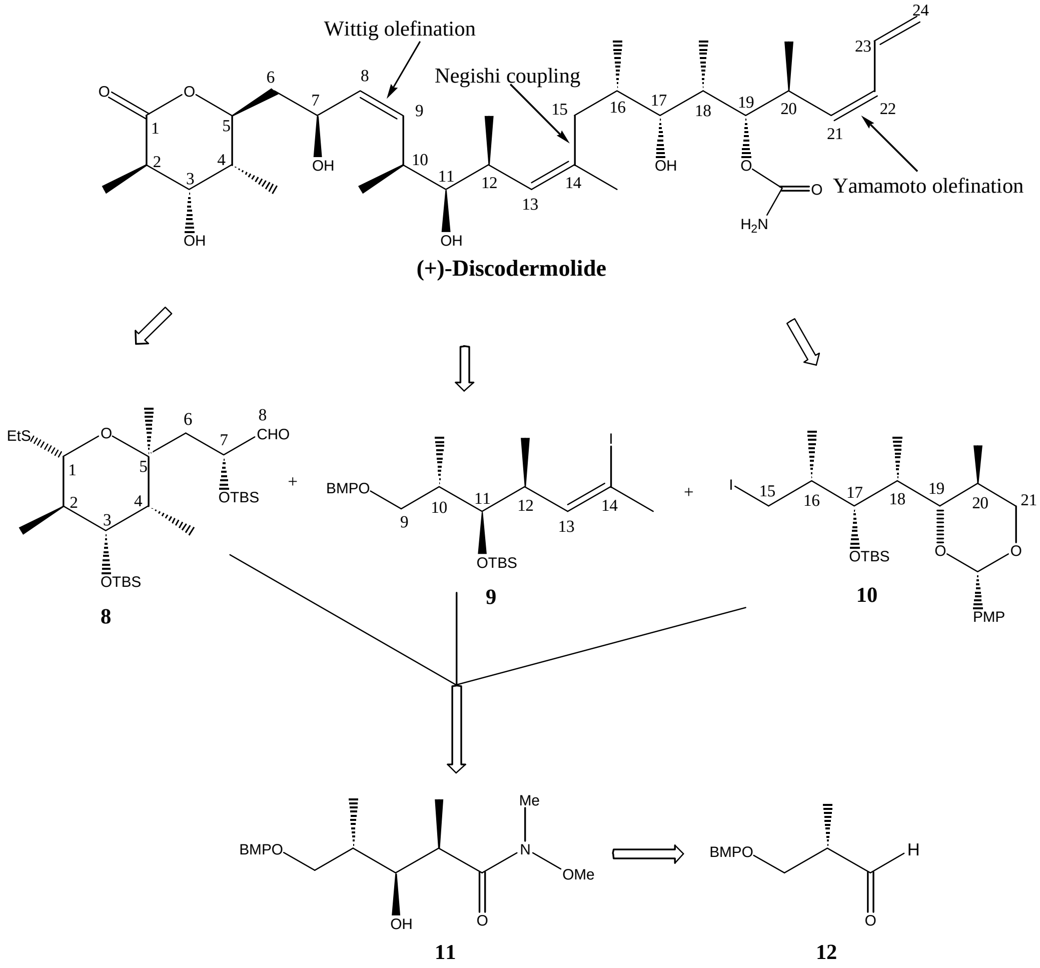

**SCHEME 2**

However, in the Smith second-generation approach, as well as in their first effort, the limiting step is the phosphonium salt **14** that was prepared from **13** at ultrahigh pressure (12.8 Kbar = 12.6 × $10^{-3}$ atm) for 6d using a specialized reactor[44](Scheme 3). Due this problem, Smith's group have developed a third-generation synthesis[36a] that does not require ultrahigh pressure to produce the Wittig salt required for construction of the (C8-C9) disubstituted olefin. This approach holds promise as an efficient, scalable, and practical synthetic route to provide the (+)-discodermolide[36a].

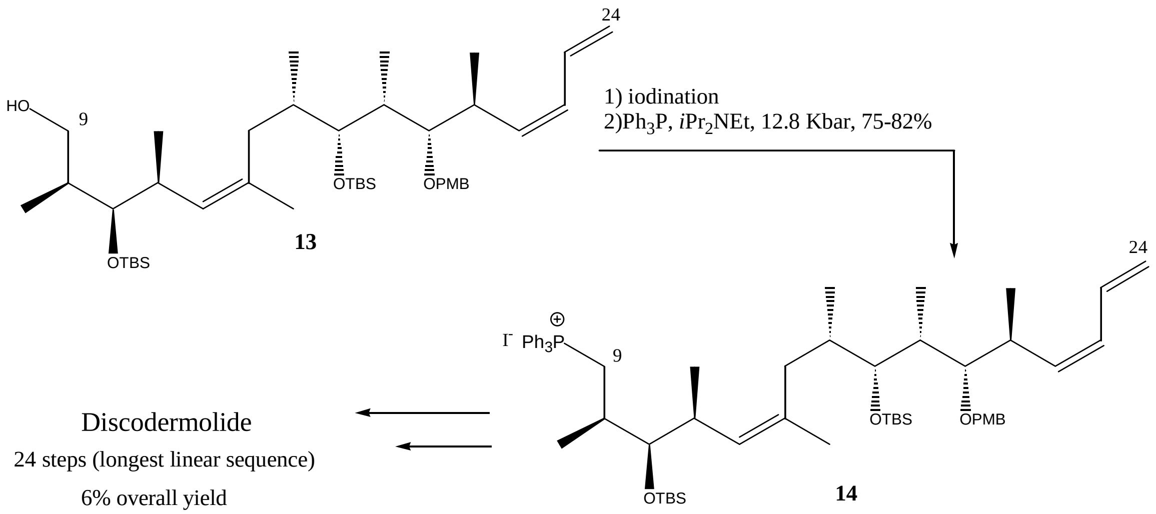

**SCHEME 3**

The Smith synthesis yields an impressive 1.043 g of discodermolide, which is more than has ever been isolated from the sponge source (7 mg from 434 g of sponge) and represents an important contribution to further biological and preclinical evaluation.

## Myles Synthesis of *ent*-Discodermolide

In the total synthesis of *ent*-discodermolide accomplished by Myles et al.[38], the three fragments **15** (C1-C7), **16** (C9-C15), and **17** (C16-C21) were employed (Scheme 4), with key couplings made at (C7-C8) based on a Nozaki-Kishi addition[43] and (C15-C16) relying on an enolate alkylation step. The Myles synthesis provided the *ent*-discodermolide with an overall yield of 1.4% from iodide **17**.

## Marshall Synthesis of Discodermolide

The synthetic strategy adopted by Marshall et al. for (+)-discodermolide[39] involved the three fragments **20** (C1-C7), **21** (C8-C13), and **22** (C15-C24), with key coupling steps accomplish at (C7-C8) by lithium acetylide addition to an aldehyde and (C14-C15) using a novel Suzuki cross-coupling reaction[45] (Scheme 5).

The fragments **20** (C1-C7) and **21** (C8-C13) were furnished with *dr* = 9:1 by the addition of a chiral allenylzinc species, prepared *in situ* by the treatment of propargylic mesylate **25** with $Et_2Zn$ and catalytic $Pd^0$, to the $\alpha$-chiral aldehyde **23**. Protection of the resulting alcohol **26** as the MOM ether gave the (C8-C13) fragment **21**. Subsequently a nine-step sequence provided the (C1-C7) fragment **20** from **26** (Scheme 6). The (C15-C24) fragment **22** was built using methodology developed within the Marshall group[46], involving the addition of chiral allenylstannane **28** to the Roche ester–derived aldehyde **6** to give **29** with *dr* = >20:1 (Scheme 7). Introduction of the C19 and C20 stereocenters utilized a sequence of reduction, Sharpless epoxidation, and methyl cuprate opening of the resulting epoxide to afford diol **30**[39b]. Transformations completed the synthesis of the iodide **22** in to a further eight steps (Scheme 7). The Marshall synthesis of discodermolide was completed in 2.2% overall yield achieved over 29 steps (longest linear sequence).

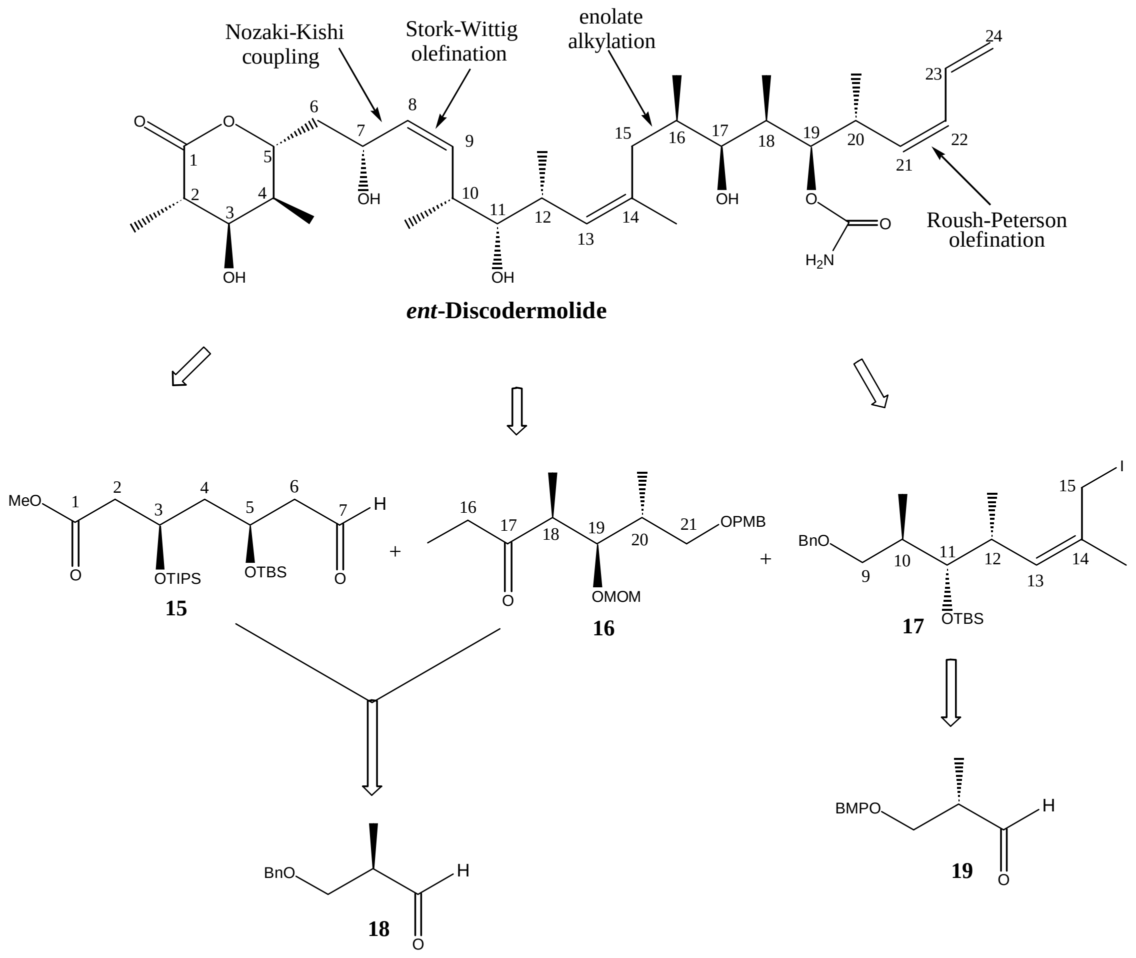

SCHEME 4

## Paterson Synthesis of Discodermolide

The professor Paterson, in addition to writing a good review of the development of a practical total synthesis of discodermolide[35], also made important contributions with the first- and second-generation total synthesis of (+)-discodermolide and structural analogs[40,42c]. In the second-generation synthesis, a revised strategy towards discodermolide was elaborated both to eliminate the use of all chiral reagents and auxiliaries, and to reduce the total number of steps[40d]. To achieve these specific goals, a novel aldol coupling across (C5-C6) was employed between aldehyde **31** and methyl ketone **32**, relying on long-range asymmetric induction from the C10 γ-stereocenter (Scheme 8). The use of a common building block **33**, containing the repeating *anti-syn* stereotriad found in the three subunits **31**, **34,** and **35**, helped to reduce the total number of steps (Scheme 8). The second-generation synthesis of discodermolide developed by Paterson's group proceeded in 5.1% yield over a 24-step longest linear sequence (35 total step). In comparison with the original route, the Paterson second-generation synthesis reduced substantially the total number of steps required to complete discodermolide and provided a more cost-effective route.

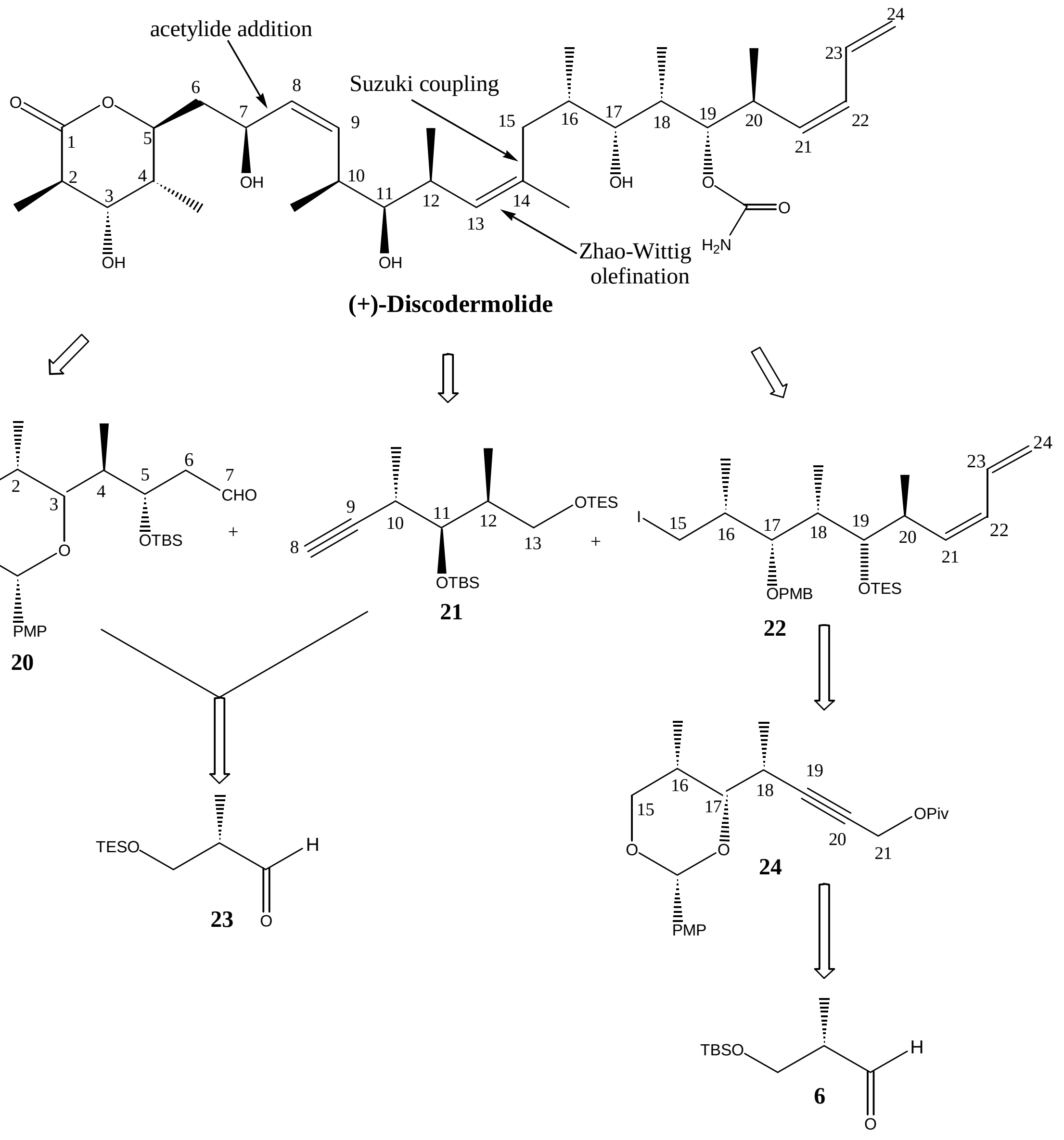

SCHEME 5

## STRUCTURE-ACTIVITY RELATIONSHIPS

The Schreiber group was the first to describe the synthesis of analogs, laying the groundwork for a more detailed SAR study[18]. The synthesis of stereoisomers of (+)-discodermolide (Fig. 5) was performed by the Schreiber group[48], which were tested for their ability to inhibit the replication of cells by using a [$^3$H]thymidine incorporation assay (Table 1). Replicating cells incorporate thymidine into their DNA during the S phase of the cell cycle. The cell growth-inhibitory properties of these compounds suggested that the configuration of C16 and C17 is critical to discodermolide's activity. Relative to (+)-discodermolide, which has an $IC_{50}$ of 6 n*M* in the [$^3$H]thymidine assay, compound **37** had an $IC_{50}$ value of 300 n*M* and compound **38** was completely inactive. In contrast, the 16-normethyl compound **39** was as active *in vivo* as (+)-discodermolide itself.

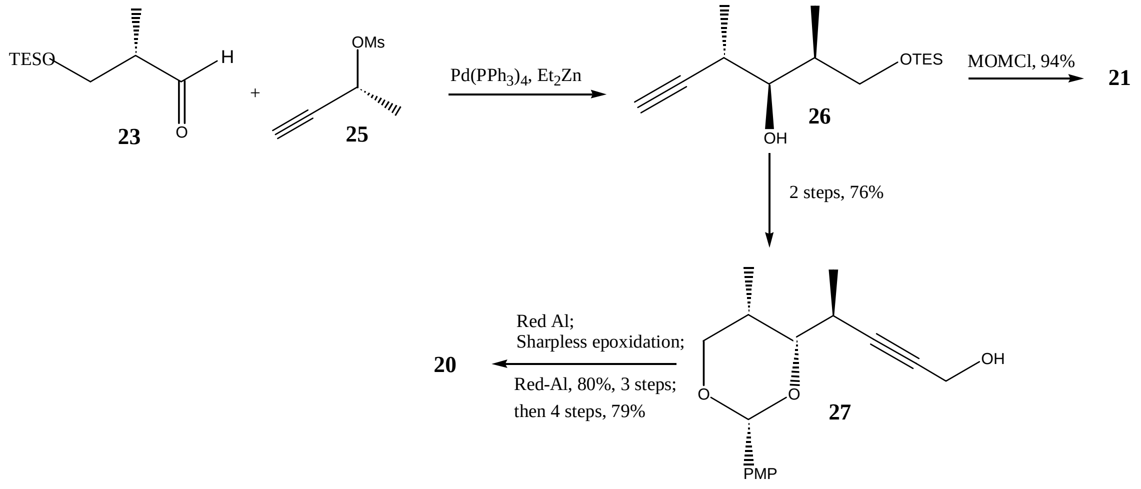

**SCHEME 6**

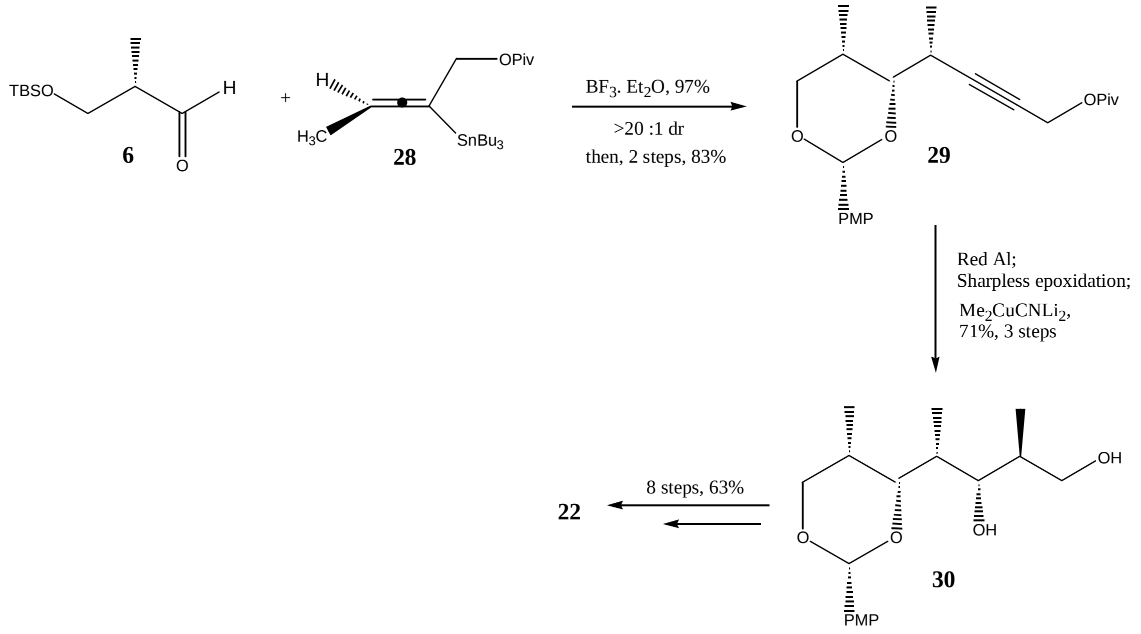

**SCHEME 7**

The Schreiber group also synthesized thiophenyl derivatives of (+)-discodermolide in the lactone ring with minimal disturbance of conformation[48]. Both α and β thiophenyl anomers, as well as the 16-desmethyl version of the β anomer, were synthesized, affording **40**, **41,** and **42** respectively (Fig. 6), to examine differences in activity due to the stereochemistry at the anomeric center. The anomers were equally active in inhibiting proliferation as natural (+)-discodermolide (Table 2).

Other fragments and analogs of (+)-discodermolide (Fig. 7) have been made and tested by the Schreiber group[48], thus acquiring useful information for SAR study. The fragments **43**, **44,** and the analog **45** of (+)-discodermolide were inactive *in vivo*, however the analogs **46** and **47** appeared promising as binding reagents and their $IC_{50}$ values were both 70 nM. These $IC_{50}$ were measured by a $[^3H]$thymidine incorporation assay using MG63 cells.

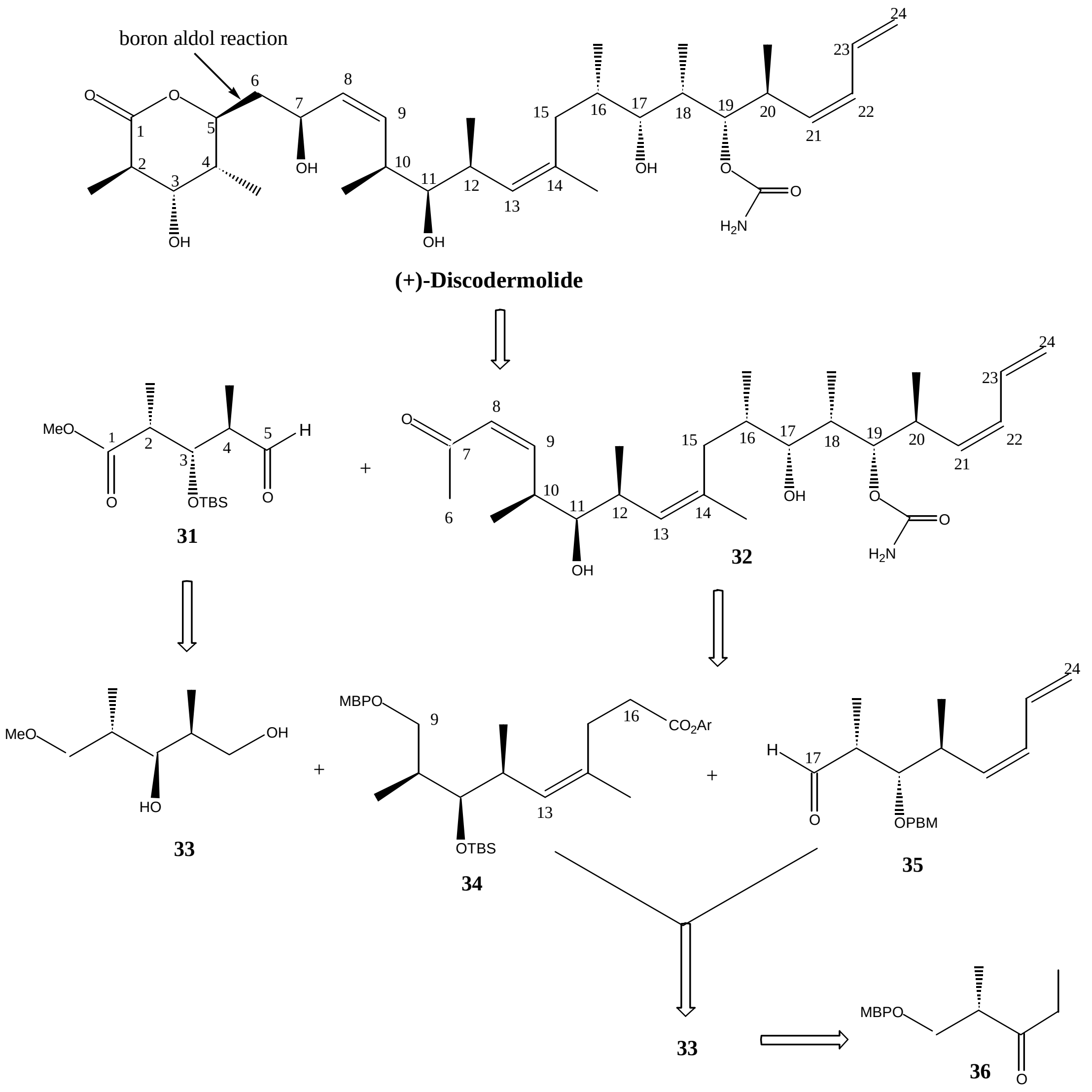

**SCHEME 8**

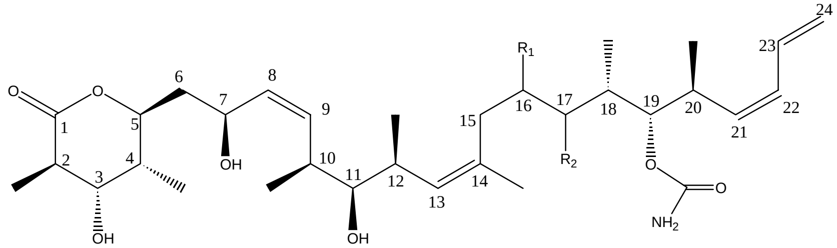

| | $R_1$ | $R_2$ |
|---|---|---|
| **37** | R-Me | R-OH |
| **38** | S-Me | S-OH |
| **39** | H | R-OH |

**FIGURE 5.** Stereoisomers of (+)-discodermolide synthesized by Schreiber et al.

**TABLE 1**
**Biological Activity of Stereoisomers of (+)-Discodermolide Determined by Schreiber et al.**

| Product | $R_1$ | $R_2$ | $IC_{50}$ |
|---|---|---|---|
| **(+)-Discodermolide** | S-Me | R-OH | 6nM |
| **37** | R-Me | R-OH | 300nM |
| **38** | S-Me | S-OH | - |
| **39** | H | R-OH | 10nM |

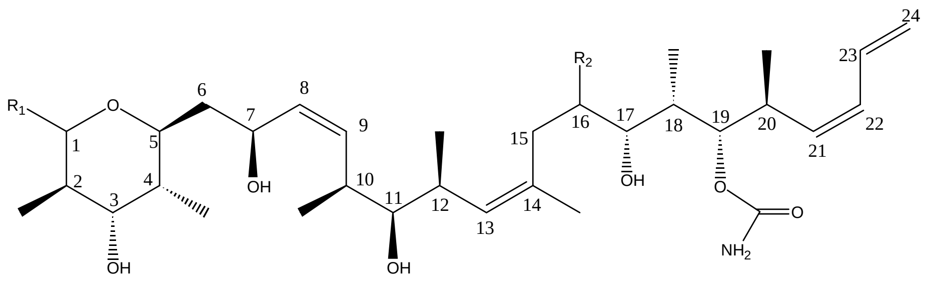

| | $R_1$ | $R_2$ |
|---|---|---|
| **40** | β–PhS | S-Me |
| **41** | α–PhS | S-Me |
| **42** | β–PhS | H |

**FIGURE 6.** Thiophenyl analogs of (+)-discodermolide synthesized by the Schreiber group.

**TABLE 2**
**Biological Activity of Thiophenyl Analogs of (+)-Discodermolide Determined by the Schreiber Group**

| Product | $R_1$ | $R_2$ | $IC_{50}$ |
|---|---|---|---|
| **40** | β–PhS | S-Me | 6nM |
| **41** | α–PhS | S-Me | 4nM |
| **42** | β–PhS | H | 4nM |

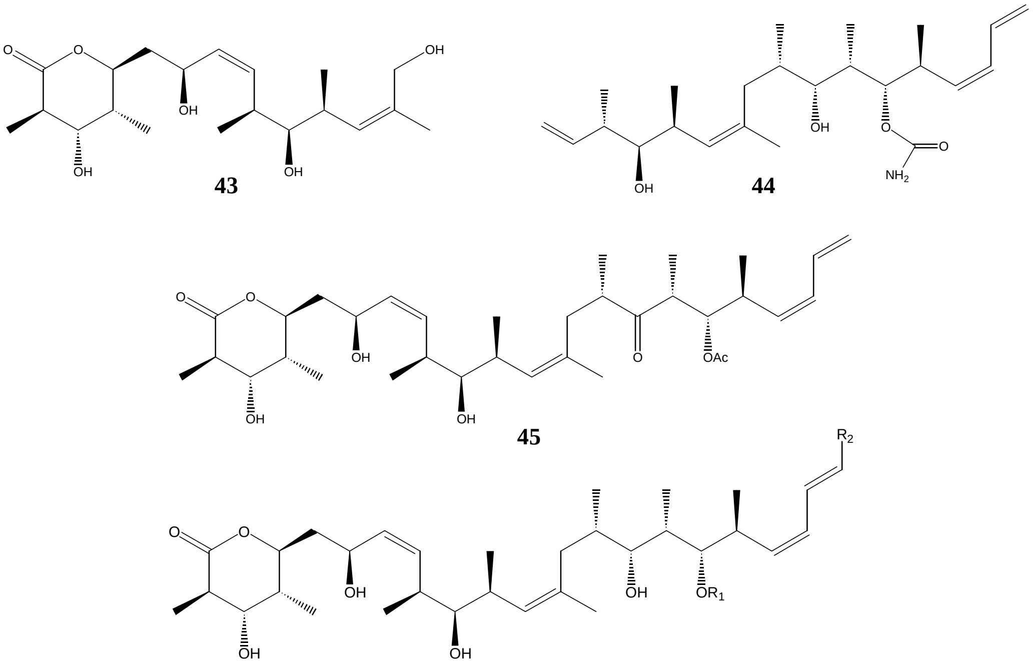

**46** $R_1$ = $CO(CH_2)_5NHFmoc$ $R_2$ = H

**47** $R_1$ = $CONH_2$ $R_2$ = $-(CH_2)_4OPv$

**FIGURE 7.** Fragments and analogs of (+)-discodermolide prepared by the Schreiber group.

Another example that could provide valuable information for a better understanding of the biological activity of (+)-discodermolide is the synthesis of three epimeric discodermolides **48–50** by the Paterson group[40a] (Fig. 8).

A study published by the Smith and Horwitz groups[49] compares the activity of (+)-discodermolide with the four new analogs (Fig. 9) synthesized *via* a highly efficient, triply convergent approach. This study provides important new information on the structural requirements responsible for the unique nucleation and potency of (+)-discodermolide. Their results[49] showed that the natural product is extremely sensitive to small modifications in the (+)-discodermolide structure (Table 3). The *in vitro* studies demonstrated that (+)-discodermolide and the structural analogs induced tubulin assembly in the absence of GTP and that the microtubules formed were stable under depolymerizing conditions. The initial rate of tubulin polymerization in the presence of (+)-discodermolide was dramatically different compared of the analogs **51–54** (Fig. 9). The microtubule protein (MTP) from each *in vitro* assay was examined by electron microscopy to confirm that normal microtubules were formed in the presence of the compounds **51–54**. An interesting observation was that small changes in the (+)-discodermolide structure, such as in analog **51**, resulted in a significant loss of the enhancement of tubulin nucleation, presumably due to a loss of a specific contact between the analogs and β-tubulin that must be necessary for enhanced initiation of tubulin polymerization. The microtubules assembled in the presence of (+)-discodermolide were approximately 5-fold, 12-fold, and 4-fold shorter than those formed by analogs **51** and **52**, by analogs **53** and **54**, and by Taxol®, respectively. In order to determine the ability of the discodermolide analogs to inhibit the binding of Taxol® to preformed microtubules, a drug-binding competition assay was performed using [$^3$H]Taxol and purified tubulin by the Smith group. It was found that each of the compounds was a competitive inhibitor of the binding of [$^3$H]Taxol to the preformed microtubules. At 1 μ*M*, Taxol®, (+)-discodermolide, and analogs **51–54** exhibited very similar inhibition, whereas analog **54** was essentially inactive in displacing [$^3$H]Taxol binding. The Horwitz group[49] also showed that (+)-

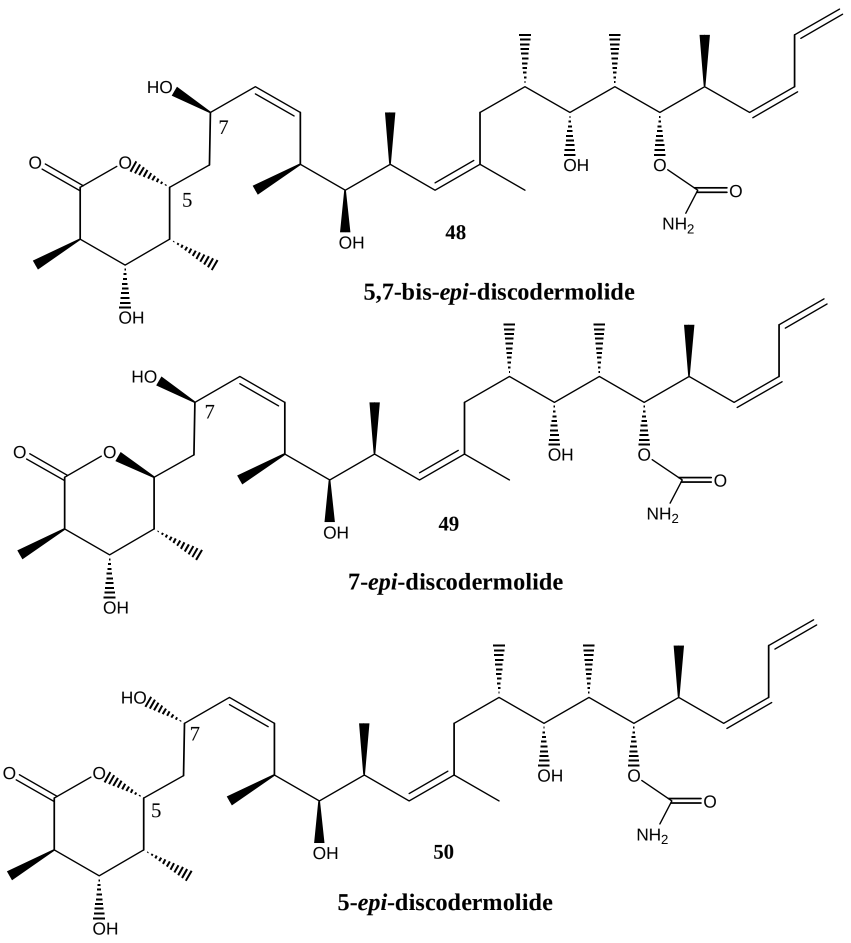

**FIGURE 8.** Three epimeric discodermolides prepared by the Paterson group.

discodermolide and the analogs all inhibited the proliferation of A549 lung cells, but to different extents. Taxol® and analog **51** shared similar $IC_{50}$ values, followed by (+)-discodermolide. Analog **52**, which was as potent *in vitro* as the above compounds, was approximately 2-fold less cytotoxic than (+)-discodermolide. Although analogs **53** and **54** shared similar *in vitro* activity, analog **53** had an approximately 3-fold decrease in cytotoxicity while analog **54** had a 128-fold decrease compared to (+)-discodermolide. Similar results were obtained with SKOV3 ovarian carcinoma cells, although (+)-discodermolide was approximately 8-fold less active in this cell line compared to A549 cells. Taxol® and analog **51** had comparable $IC_{50}$ values, while (+)-discodermolide, analog **52** and analog **53** were less cytotoxic. As seen in A549 and SKOV3 cells (Table 3), analog **54** displayed the greatest decrease in cytotoxicity due to a change in the olefin geometry at C8 position.

The semisynthesis of new discodermolide analogs (Fig. 10) prepared from the natural product also helps to understand the biological activity and the SAR of (+)-discodermolide. A good example is the semisynthesis done by the Gunasekera group[50] who prepared eight acetylated analogs of (+)-discodermolide and evaluated their *in vitro* cytotoxicity against murine P-388 leukemia cells. The acetylated analogs were prepared by treating natural (+)-discodermolide in dry pyridine with acetic anhydride at a controlled temperature of around 15°C. The mixture was separated by HPLC to give eight discodermolide analogs. These analogs inhibited the *in vitro* proliferation of cultured murine leukemia cells with $IC_{50}$ values of (+)-discodermolide, 35; **55**, 837; **56**, >6925; **57**, 166; **58**, 0.74; **59**, 103; **60**, 1149; **61**, 12.6; and **62**, 3.9 n*M* (Fig. 10). The biological tests obtained by the Gunasekera group[50] showed

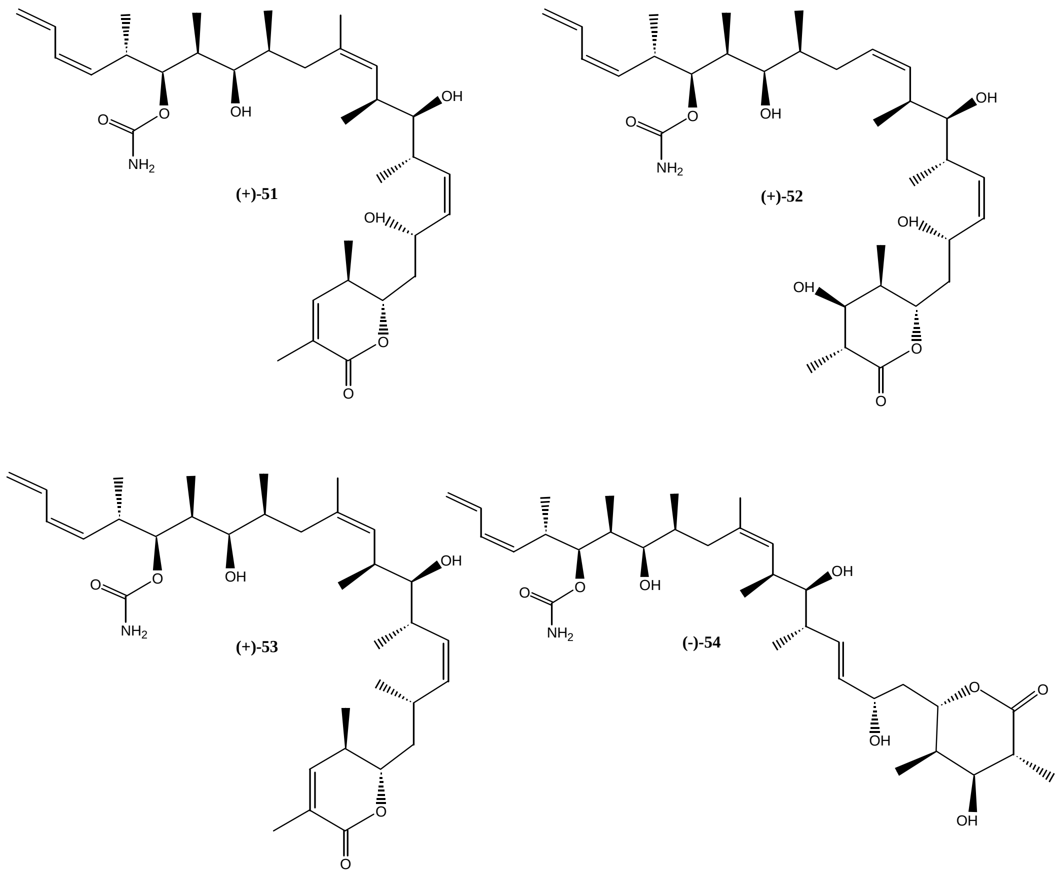

**FIGURE 9.** Analogs of (+)-discodermolide synthesized by the Smith group.

**TABLE 3**
**Biological Activity of Analogs of (+)-Discodermolide Determined by the Smith III Group**

| Compound | $IC_{50}$ | |
|---|---|---|
| | A549 | SKOV3 |
| Taxol | 1.4 | 3.3 |
| (+)-Discodermolide | 3.8 | 31.3 |
| 51 | 1.8 | 6.1 |
| 52 | 7.8 | 22.0 |
| 52 | 11.4 | 31.3 |
| 54 | 485.0 | 353.0 |

$IC_{50,}$ drug concentration that inhibits cell division by 50% after 72h.

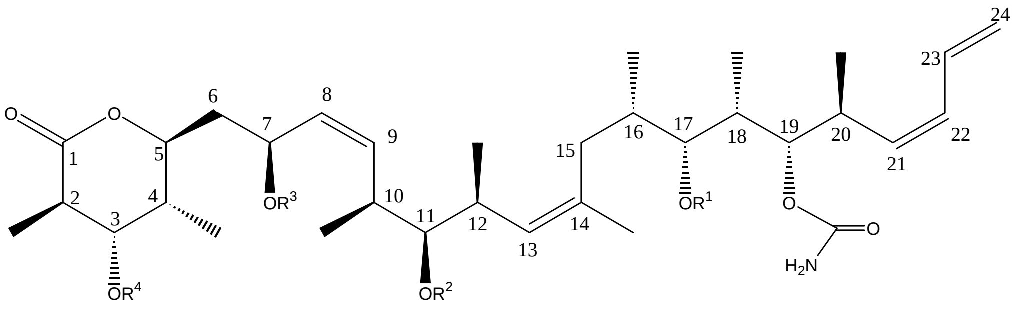

| Compound | $R^1$ | $R^2$ | $R^3$ | $R^4$ | $IC_{50}$ |
|---|---|---|---|---|---|
| **(+)-Discodermolide** | H | H | H | H | 35 |
| **55** | Ac | Ac | Ac | Ac | 837 |
| **56** | Ac | H | Ac | Ac | >6925 |
| **57** | H | Ac | Ac | Ac | 166 |
| **58** | H | H | Ac | Ac | 0.74 |
| **59** | H | Ac | H | Ac | 103 |
| **60** | Ac | H | H | Ac | 1149 |
| **61** | H | H | H | Ac | 12.6 |
| **62** | H | H | Ac | H | 3.9 |

**FIGURE 10.** Acetylated analogs of (+)-discodermolide prepared by Gunasekera et al.

that the acetylation of analogs on the left-hand side of the molecule, at position C-3 and C-7, confers a greater cytotoxicity to the discodermolide structure as seen with discodermolide-3-acetate **61**, discodermolide-7-acetate **62**, and discodermolide-3,7-diacetate **58**. It is interesting to note that analogs with acetyl groups at position C-11, discodermolide-3,11-diacetate **59,** and discodermolide-3,7,11-triacetate **57**, showed a reduced cytotoxicity compared with the parent molecule, whereas compounds that include an acetylation at position C-17 cause a dramatic reduction in the activity of the specific analogs, as seen with discodermolide-3,7,11,17-tetraacetate **55**, discodermolide-3,7,17-triacetate **56**, and discodermolide-3,17-diacetate **60**. These results are in agreement with the Schreiber group[47], who reported a 10-fold reduction in the activity of discodermolide-17-acetate as compared to (+)-discodermolide in [$^3$H]thymidine incorporation assay using MG63 cells.

The discovery of new natural discodermolide analogs from marine sponges could provide important information about the SAR of (+)-discodermolide. For example, the five new analogs trivially named 2-*epi*-discodermolide **63**, 2-*des*-methyldiscodermolide **64**, 5-hydroxymethyldisco-dermolate **65**, 19-*des*-aminocarbonyldiscodermolide **66**, and 9(13)-cyclodiscodermolide **67** (Fig. 11) have been isolated by the Gunasekera group[51] from marine sponges belonging to the genus *Discodermolide* from the Caribbean Sea. The natural analogs of (+)-discodermolide were tested for their *in vitro* cytotoxicity to cultured murine P-388 leukemia and human lung adenocarcinoma A-549 cell lines. These compounds inhibited the *in vitro* proliferation of the P-388 cell line, with $IC_{50}$ values of (+)-discodermolide, 35; **63**, 134; **64**, 172; **65**, 65.8; **66**, 128; and **67**, 5043 n*M* and the A-549 cell line, with $IC_{50}$ values of (+)-discodermolide, 13.5; **63**, 67; **64**, 120; **65**, 74; **66** and **67**; 4487 n*M*[51]. This activity data indicated that changes in the δ-lactone ring, more specifically at $C_1$ (opened lactone, **66**) and $C_{25}$ (methyl group, **63**, **64**), made only a minor contribution toward the activity. Similarly, any changes at the tail end of the molecule (carbamate group, **65**) made no significant decrease in activity. It is worth noting that changes in the middle section of the

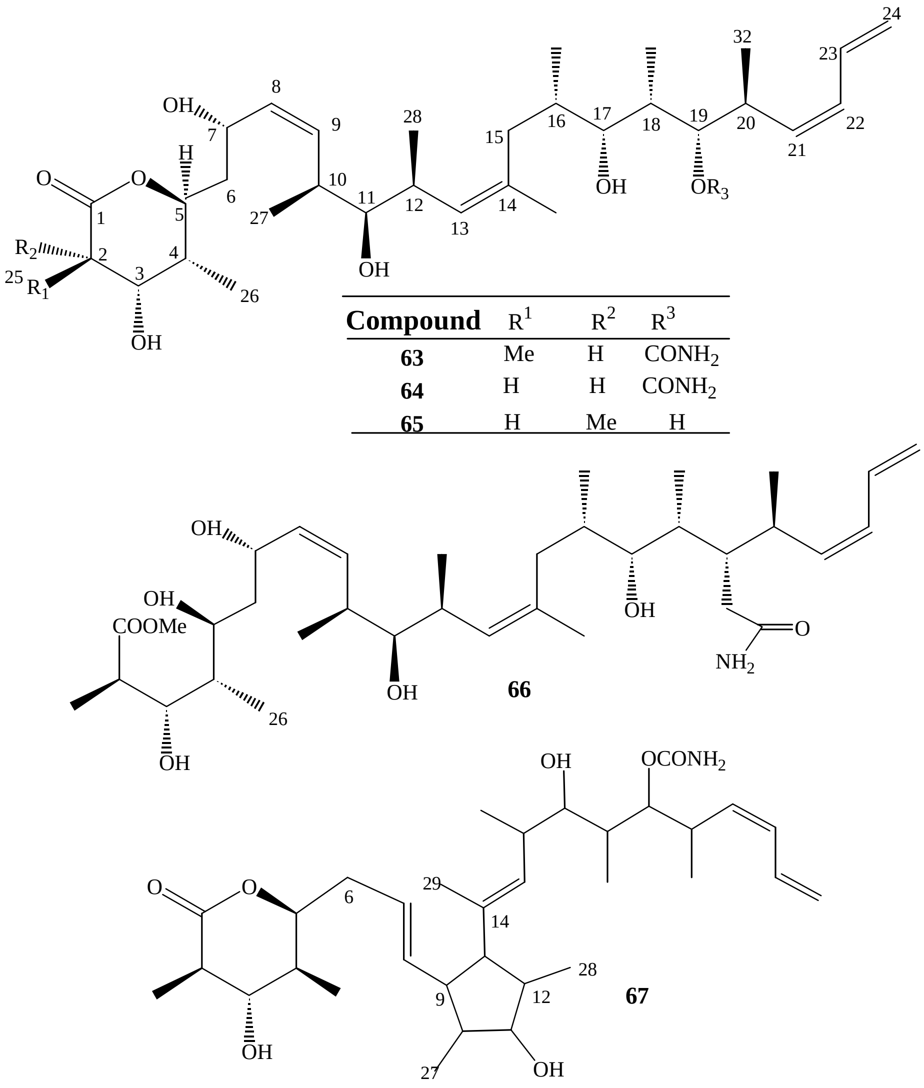

| Compound | R1 | R2 | R3 |
|---|---|---|---|
| **63** | Me | H | $CONH_2$ |
| **64** | H | H | $CONH_2$ |
| **65** | H | Me | H |

**FIGURE 11.** New natural discodermolide analogs isolated from marine sponges.

molecule **67** caused a complete loss of activity. These results are consistent with other results found previously by the Gunasekera group[17b] and suggest the importance of the $C_7$ through $C_{17}$ moiety for potency against cultured tumor cell lines.

## CONCLUSION

In recent years, natural products have been growing in importance as new antimitotic compounds that target the tubulin/microtubule system, which plays an important role in the cell cycle, particularly during mitosis. (+)-Discodermolide, a promising marine natural product that stabilized microtubules, is a good example and represents a leading compound for a new class of microtubule-stabilizing anticancer-drugs. Although (+)-discodermolide is one of several bioactive molecules that are naturally produced by marine organisms, supplies of these compounds are only available in limited quantities from their natural sources. Due to the demands of the pharmaceutical industry for (+)-discodermolide, researchers followed up on the discovery by focusing on ways to create an alternative for producing this natural product. One good alternative is to be found in total synthesis that is able to produce useful quantities, as well as synthetic analogs of discodermolide, which may lead to a better understanding of the structure-activity relationship (SAR) and allow the development of a drug that could be more potent and easier to synthesize than the parent compound, (+)-discodermolide.

## ABBREVIATIONS

Ac: acetyl; Ar: unspecified aryl group; Bn: benzyl; Bu: butyl Bz: benzoyl; *dr*: diastereomeric ratio; Et: Ethyl; Me: Methyl; MOM: methoxymethyl; Ms: methylsulfonyl; MTP: microtubule protein; Piv: pivaloyl; PMB: *p*-methoxybenzyl; PMP: *p*-methoxyphenyl; Pr: propyl; TBS: *tert*-butyldimethylsilyl; TES: triethylsilyl.
